# 66958 Team science training in an engineering design program improves psychological safety and self-efficacy within interdisciplinary teams

**DOI:** 10.1017/cts.2021.586

**Published:** 2021-03-30

**Authors:** Erin Blakeney, Soyoung Kang, Nicole Summerside, Jonathan Liu, Eric Siebel, Brenda Zierler, Jonathan Posner

## Abstract

ABSTRACT IMPACT: This project successfully implemented a promising team science model by introducing and facilitating best practices to develop high functioning teams working to accelerate health innovations from bench to bedside. OBJECTIVES/GOALS: The goal of this project was to improve the team science knowledge, skills, and attitudes of interdisciplinary engineering students (undergraduate and graduate) who were partnered with health professionals to develop technical solutions to translational health challenges during a year-long Engineering Innovation in Health (EIH) program. METHODS/STUDY POPULATION: We adapted, implemented, and evaluated team science training content and approaches in the EIH program at the University of Washington (UW). EIH faculty and the UW Institute of Translational Health Sciences’ (ITHS) Team Science Core co-developed and delivered highly interactive team science training modules and evaluated their impact with biannual surveys. A student cohort was surveyed prior to the implementation of the team science trainings, which served as a baseline. Descriptive statistics were used to summarize student demographics and survey responses within and between years. Median and interquartile range of responses to Likert-type questions were calculated, and Mann-Whitney U Tests (independent samples Wilcoxin Rank Sum Tests) were used to test for differences within and between years. RESULTS/ANTICIPATED RESULTS: During both the baseline and the team training year, student demographics were similar in terms of gender and past experience working in teams. Team training during the first year of implementation was well-received. Post-implementation surveys of students demonstrated measurable improvement in team dynamics, communication, and effectiveness; including, students reporting higher levels of psychological safety and self-efficacy within their teams. Comparisons within the team training year and between the baseline and team training years identified numerous instances in which differences were statistically significant. DISCUSSION/SIGNIFICANCE OF FINDINGS: Tailored team science training in an interdisciplinary EIH program was successful at improving psychological safety and self-efficacy among undergraduate and graduate students and offers a promising model for similar settings and audiences.

